# Comparative Chloroplast Genomes of Four *Lycoris* Species (Amaryllidaceae) Provides New Insight into Interspecific Relationship and Phylogeny

**DOI:** 10.3390/biology10080715

**Published:** 2021-07-27

**Authors:** Fengjiao Zhang, Ning Wang, Guanghao Cheng, Xiaochun Shu, Tao Wang, Weibing Zhuang, Ruisen Lu, Zhong Wang

**Affiliations:** 1 Jiangsu Key Laboratory for the Research and Utilization of Plant Resources, Institute of Botany, Jiangsu Province and Chinese Academy of Sciences (Nanjing Botanical Garden Mem. Sun Yat-Sen), Nanjing 210014, China; fengjiao@cnbg.net (F.Z.); ningw813@163.com (N.W.); chengguanghao520@163.com (G.C.); islbe@163.com (X.S.); johnwt1007@163.com (T.W.); weibingzhuang@cnbg.net (W.Z.); 2 The Jiangsu Provincial Platform for Conservation and Utilization of Agricultural Germplasm, Institute of Botany, Jiangsu Province and Chinese Academy of Sciences (Nanjing Botanical Garden Mem. Sun Yat-Sen), Nanjing 210014, China

**Keywords:** *Lycoris*, Amaryllidaceae, chloroplast genome, interspecific comparison, phylogenetic analysis

## Abstract

**Simple Summary:**

The genus *Lycoris* (Amaryllidaceae) comprises about 20 species with high ornamental and medicinal value. However, germplasm identification is still difficult due to frequent interspecific hybridization and intraspecific morphological variation within this genus. Plastid genome sequencing has been proven to be a useful tool to identify closely related species and is widely used in the field of plant evolution and phylogeny. In the present study, we provided four chloroplast genomes of *Lycoris* and retrieved seven published species in the genus for comparative genomics and phylogenetic analyses. All these chloroplast genomes possess the typical quadripartite structure with conserved genome arrangement and gene content, yet their lengths varied due to expansion/contraction of the IR/SC boundaries. Phylogenetic relationships within *Lycoris* were resolved with high resolution using complete cp genome sequences. These results could not only offer a genome-scale platform for identification and utilization of *Lycoris* but also provide a phylogenomic framework for future studies in this genus.

**Abstract:**

The genus *Lycoris* (Amaryllidaceae) consists of about 20 species, which is endemic to East Asia. Although the *Lycoris* species is of great horticultural and medical importance, challenges in accurate species identification persist due to frequent natural hybridization and large-scale intraspecific variation. In this study, we sequenced chloroplast genomes of four *Lycoris* species and retrieved seven published chloroplast (cp) genome sequences in this genus for comparative genomic and phylogenetic analyses. The cp genomes of these four newly sequenced species were found to be 158,405–158,498 bp with the same GC content of 37.8%. The structure of the genomes exhibited the typical quadripartite structure with conserved gene order and content. A total of 113 genes (20 duplicated) were identified, including 79 protein-coding genes (PCGs), 30 tRNAs, and 4 rRNAs. Phylogenetic analysis showed that the 11 species were clustered into three main groups, and *L. sprengeri* locate at the base of *Lycoriss*. The *L. radiata* was suggested to be the female donor of the *L. incarnata, L. shaanxiensis*, and *L. squamigera*. The *L. straminea* and *L. houdyshelii* may be derived from *L. anhuiensis*, *L. chinensis*, or *L. longituba*. These results could not only offer a genome-scale platform for identification and utilization of *Lycoris* but also provide a phylogenomic framework for future studies in this genus.

## 1. Introduction

The genus *Lycoris* Herb. is a group of perennial bulbous plants with high ornamental and medicinal values that belongs to the family Amaryllidaceae [1,2]. More than 110 Amaryllidaceae alkaloids were identified in *Lycoris*, which have the function of antitumor, antibacterial, cytotoxic, and cholinesterase inhibition activities [3]. Species of *Lycoris* are widely cultivated as ornamental plants for their large and beautiful flowers [4].

The genus contains about 20 species, mainly distributed in China (15 species recorded) and Japan, and a few in Myanmar and North Korea. It has been demonstrated that frequent interspecific hybridization and intraspecific morphological variation commonly happen in *Lycoris* [5], resulting in the difficulty to make a clear standard for germplasm identification at the morphological level. Moreover, new *Lycoris* species are being reported, such as, *L. hunanensis* which was published as a new species from Yuanling County in China, which showed some difference from *L. straminea* [6]. *Lycoris* × *hubeiensis* K. Liu was identified as a natural hybrid of putative parents *L. radiata* and *L. aurea* [7]. In the Tsinling Mountains in China, *L. tsinlingensis* was found and published as a new species, but it is largely similar to *L. chinensis* [8]. In fact, there are different opinions on whether these subtle differences could be used as a criterion for determining a new species, so a feasible evaluation standard was suggested to clarify whether it is a new species or a variant. It requires some specific sequences for germplasm identification and a clear interspecific relationship in *Lycoris*.

To explore the interspecific relationships and clarify the hypothesis of hybrid origin in *Lycoris*, molecular markers of RAPD (random amplified polymorphic DNA) [9], nuclear ITS (internal transcribed spacer) sequences [10], inter-simple sequence repeat (ISSR) [11], and SCoT (start codon targeted) [12] have been used. RAPD analysis was consistent with the classification based on chromosome karyotype, which divided 13 *Lycoris* species into two groups. Nuclear ITS sequences of 15 *Lycoris* species suggested the three infrageneric clades and the hybrid origin of *L. straminea*, *L. caldwellii*, and *L. albiflora*. However, the extensive sequence variation has existed in many plant genomes, the complex and unpredictable evolutionary behavior of ITS sequence reduced the utility for phylogenetic analysis [13]. Thus, more methods were developed, inter-simple sequence repeat (ISSR) analyses of 20 species and varieties indicated a high level of genetic variation among species in *Lycoris*, and four major groups clustered by UPGMA analysis presented a consistence with morphological and karyotype observations. SCoT markers of 14 *Lycoris* species were tested and clustered into four groups, in which *L. squamigera*, *L. incarnata*, and all hybrids with the characteristic of multi-colored flowers were gathered together, suggesting the possibility of the hybrid origin of these two species. Although several strategies were developed for the analysis of interspecific relationships in the genus *Lycoris*, each method offered limited resolution within closely related species, resulting in that they did not get unanimous conclusion. More effective molecular markers are needed to be developed for germplasm identification, conservation, utilization, and breeding of the *Lycoris* species.

Plastid genes are regularly utilized in biotechnology or phylogeny, but with the limitation of DNA sequencing costs, investigators always chose a dense taxon sampling, which had a small number of informative loci for molecular phylogenetic analysis in *Lycoris*. For example, the cpDNA *trnL-F* sequence of 15 *Lycoris* species was selected to construct a phylogeny tree, which contained three infrageneric clades and was basically consistent with the classification of morphology except for *L. longituba*, *L. aurea*, and *L. straminea*. Phylogenetic reconstruction was obtained using plastid markers (*trnS-trnfM* and *trnC-ycf6*), which clustered *Lycoris* spp. into three clades and differed from that derived using ITS sequences [14]. Considering the rapid radiations and conservative genome evolution, limited sequence variation could be detected, particularly at low taxonomic levels. More sequence information and species were often desirable to increase phylogenetic resolution. Actually, complete chloroplast genome sequences were more highly discriminating and efficient as plant DNA barcodes. The development of next-generation DNA sequencing has brought the benefits of large numbers of genome data collection and allowed the rapid obtaining of complete organellar genomes. Whole plastome sequencing has been an efficient option to increase the phylogenetic resolution for the phylogenetic analyses, especially at lower taxonomic levels [15,16]. In angiosperms, the cp genome is highly conserved in terms of structure, content, and order of genes [17]. They usually have a circular structure, where two large, inverted repeat (IR) regions were separated by a large single-copy (LSC) region and a small single-copy (SSC) region [18]. The cp genome sequences contain many noncoding and variation regions, which has provided an essential molecular source for interspecific phylogenetic and phylogeographic studies [19]. It has been successfully used in many families and genus, for example, *Dracunculus* (Araceae), *Cardiocrinum* and *Amana* (Liliaceae) [20,21], *Artemisia* (Asteraceae) [22], and *Withania somnifera* (Solanaceae) [23].

Since the first *Lycoris* complete cp genome of *L. squamigera* was published in 2018 [24], there are six *Lycoris* species that have been published, and a phylogenetic tree based on the complete cp genome sequences was constructed [25]. There is no doubt that more complete chloroplast genome sequences will provide more information and insights for phylogenetic relationship reconstruction. In this study, we sequenced complete cp genomes of four *Lycoris* species. Based on previous studies, we systematically analyzed the similarities and differences of global structural patterns, variations of genes, simple sequence repeats (SSRs), and inverted repeats. Then the phylogenetic relationship was constructed based on the complete chloroplast genome sequences of 11 species *Lycoris*. The comparative analysis has demonstrated the effectiveness and applicability of chloroplast genome sequences for *Lycoris* phylogeny and remarked on the potential applications for species identification, development of DNA barcoding, and future phylogenetic studies of the genus and family.

## 2. Materials and Methods

### 2.1. Plant Sample Collection, DNA Extraction and Sequencing

The bulbs of *L. incarnata*, *L. shaanxiensis*, *L. straminea*, and *L. houdyshelii* were planted in Nanjing Botanical Garden, Mem. Sun Yat-sen (E118_83, N32_06), Nanjing, China. The specimen of *L. incarnata* (No. SYS00024942) was stored at the herbarium of Sun Yat-Sen University, the specimen of *L. straminea* (No. 00110652) and *L. houdyshelii* (No. 00110525) were stored at the herbarium of the Institute of Botany, Chinese Academy of Sciences. The *L. shaanxiensis* was collected from Shanxi Province and identified in 2018, but there was no specimen record in the herbarium in China. Fresh leaves were collected, quick freezed in liquid nitrogen, then stored at −80 °C until use. Genomic DNA was extracted using the Plant Genomic DNA Kit (Huayueyang, Beijing, China). DNA integrity was examined by electrophoresis in 1% (*w*/*v*) agarose gel, and concentration was measured using a NanoDrop spectrophotometer 2000 (Thermo Scientific; Waltham, MA, USA), then accurate quantifications were completed by Qubit 2.0. High-quality DNA libraries were constructed and sequenced at Novogene Bioinformatics Technology Co., Ltd. (https://www.novogene.com/, accessed on March 2011 Tianjin, China). The strategy of Nova-PE150 was selected for high-throughput sequencing, with an insert size of 350 bp.

### 2.2. Complete Cp Genome Assembly, Annotation and Structure Analysis

The complete cp genomes were assembled using the organelle assembler NOVOPlasty (Version 3.3) [26] with the parameters of genome range (148,500–168,500) and k-mer (39). The complete cp genome sequence of *L. radiata* (GenBank accession no. MN158120) was set as a reference [27]. Assembled genome sequences were manually corrected by BLASTn comparison and circularized. GC content was calculated by Geneious software (version R11, http://www.geneious.com accessed on 3 October 2017). Correct cp genome sequences were input on web server CPGAVAS2 (http://www.herbalgenomics.org/cpgavas2, accessed on 14 October 2020.) for the cp genome annotation and visualization with the default parameters. Microsatellite sequences were identified with MISA [28], which set the unit_size/min_repeats as 1/10, 2/6, 3/5, 4/5, 5/5, and 6/5. The maximum length of a sequence between two SSRs was set as 100. MEGA [29] was performed for calculating the relative synonymous codon usage (RSCU) values. Seven previously reported *Lycoris* chloroplast genome sequences, i.e., *L. squamigera* (MH118290), *L. radiata* (MN158120), *L. sprengeri* (MN158986), *L. longituba* (MN096601), *L. chinensis* (MT700549), *L. anhuiensis* (MT700550, and *L. aurea* (NC_046752), were downloaded from the National Center of Biotechnology Information (NCBI) database. The obtained cp genome sequences in the present study were deposited in the NCBI, with the GenBank accession numbers of MW477439 (*L. incarnata*), MW477440 (*L. shaanxiensis*), MW477441 (*L. straminea*), and MW477442 (*L. houdyshelii*).

### 2.3. Interspecific Comparison of Chloroplast Genomes

To explore the divergence regions in *Lycoris*, the program IRscope (https://irscope.shinyapps.io/irapp/, accessed on 20 June 2021) was used to visualize the divergence on the boundaries of the junction sites of the 11 chloroplast genome sequences in *Lycoris* [30]. The mVISTA program (http://genome.lbl.gov/vista/index.shtml, accessed on 23 June 2021) [31] was used to align and compare the complete cp genomes of *Lycoris* with the default parameters. Each annotation of the *Lycoris* species was selected as a reference, and Shuffle-LAGAN mode was visualized in an mVISTA plot.

### 2.4. Phylogenetic Analyses

A total of 11 complete cp genome sequences of *Lycoris* were used for phylogenetic analysis, including seven reported species and four new species in this study, *Narcissus poeticus* (MH706763) was selected as outgroup taxa. The reported sequences were downloaded from the NCBI database. Both the complete plastid sequences and 79 common PCGs (output by Geneious) were used for the ML tree construction. The nucleotide sequences were aligned using the MAFFT plugin [32,33] in Geneious with default settings. All gaps are treated as missing data. The complete alignment was used to reconstruct a maximum likelihood tree using PHYML [34] with 1000 bootstrap replicates. The GTR+G+I model suggested by jModelTest 2.1.4 [35] was used for each dataset.

## 3. Results and Discussion

### 3.1. General Features of the Cp Genomes of Lycoris

**Figure 1:**
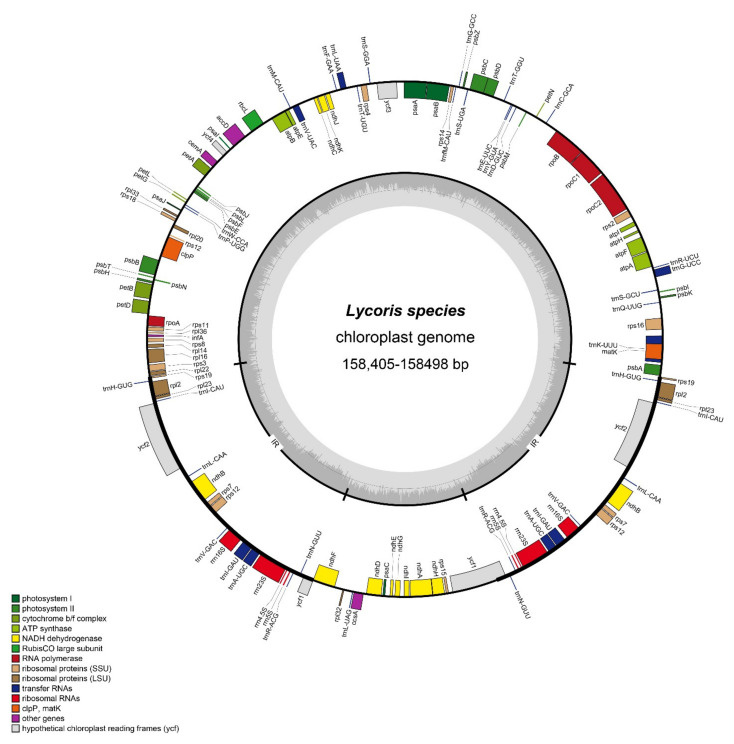
The plastome features of the *Lycoris* species. The map contains four rings, from the center going outward, the first circle means the forward and reverse repeats connected with red and green arcs, respectively. The second circle shows the tandem repeats marked with short bars. The third circle shows the microsatellites. The fourth circle shows the gene structure on the plastome. The genes were colored based on the functional categories, which were shown in the center of the map.

In the present study, we obtained four cp genomes of *Lycoris* by next-generation sequencing and de novo assembly, which were *L. incarnata*, *L. shaanxiensis*, *L. straminea*, and *L. houdyshelii*. A total of 7.2, 6.7, 5.5, and 4.8 million reads were obtained, and the average organelle coverage reached 7291×, 6761×, 5531× and 4581×, respectively (Table 1). The complete chloroplast genomes typically range from 120 to 170 kilobase pairs (kb) in length [17]. In *Lycoris*, the length of most species was around 158 kb [25]. In the present study, the full length of four species was 158,405, 158,498, 158,490, and 158,490 bp, respectively, with the same GC content of 37.8% (Table 1). The visualized circular map showed the typical angiosperm cp genome structure in *Lycoris*, which consists of one large single-copy (86,464–86,593 bp) and one small single-copy (18,352–18,499 bp) region, separated by a pair of inverted repeat (IR) (26,730–26,765) regions (Table 1 and Figure 1).

Compared with reported *Lycoris* species [25], the complete cp genomes size ranged from 158,335 (*L. radiata*) to 158,687 bp (*L. sprengeri*). Here, the genome size of these three species is between the longest and shortest; the length of LSC and SSC regions made a greater contribution to the full size. On the contrary, the IR regions were relatively conservative. In *Lycoris* cp genomes, a total of 113 genes were annotated, including 79 protein-coding genes (PCGs), 30 tRNAs, and 4 rRNAs (Table 1 and Figure 1). Compared with our previous reports, we found that the number of genes was highly conservative in *Lycoris* cp genomes. They were divided into four categories; contained genes for photosynthesis, self-replication, other genes, and function unknown (Table 2). There were 20 genes that were duplicated more than once. Four rRNAs were duplicated, which is consistent with other *Lycoris* species [25] and most plants, such as *Allium* [36] and *Amomum* [37].

Group II (G2) introns are self-splicing RNAs and mobile elements, which could provide rich characters for comparative analysis and phylogeny construction at both infrageneric and intrafamilial levels [38,39,40]. For example, the *matK* open reading frame (ORF) has been used as a marker for plant evolutionary studies. Tnterestingly, trnK-UUU contains a group II intron (trnKI1), which encodes the *matK* ORF, which attracts interest because it represents an unusual form of a group II intron [41]. In four *Lycoris* species, there were 18 splitting genes in *L. incarnata* and 17 in the other three species. There is one more *ndhF* located in IR and SSC regions in *L. incarnata*, which happened in *L. radiata* and *L. sprengeri* [25]. Most of the splitting genes contain one intron and two exons, except for *ycf3* and *clpP*; they contained two introns and three exons (Table S1).

### 3.2. CpSSRs and Repeat Structures

**Figure 2:**
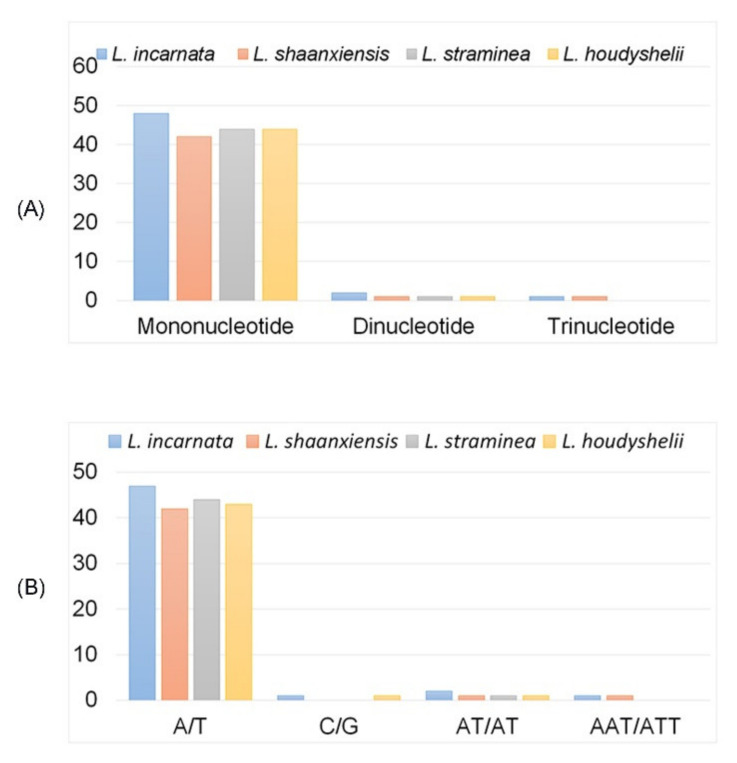
Statistics of simple sequence repeats of cp genomes of four *Lycoris* species. (**A**) Numbers of different repeat types; (**B**) Numbers of identified each SSRs motifs.

Chloroplast simple sequence repeats (cpSSRs) are microsatellites, showing typically mononucleotide tandem repeats. They commonly showed intraspecific variation in repeat numbers when they were located in the noncoding regions of the chloroplast genome [42]. Some works have proved the potential applications of variations in the noncoding regions of the chloroplast genome for phylogenetic analysis at the level of genus and species [43,44]. In the cp genomes of *L. incarnata*, *L. shaanxiensis*, *L. straminea*, and *L. houdyshelii*, there were 51, 44, 45, and 45 SSRs, respectively. The same as other reported *Lycoris* species [25], type of mononucleotide (A/T) was the most variable, which was 48, 42, 44, and 44 in four species (Figure 2 and Table S2). One mononucleotide (C/G) was detected in only *L. incarnata* and *L. houdyshelii*. One dinucleotide (AT/AT) was commonly detected in *Lycoris* species except for the *L. incarnata*, which contains two SSRs of AT/AT. A previous study also showed one dinucleotide (AT/AT) in seven *Lycoris* species [25], suggesting the conservation of dinucleotide SSRs among species. Trinucleotide (AAT/ATT) was only detected in *L. incarnata* and *L. shaanxiensis.* The trinucleotide (ATT) was only detected in *L. incarnata* and *L. shaanxiensis*, which was existed in other four species (Figure 2), *L. aurea*, *L. radiata*, *L. sprengeri*, and *L. squamigera* [25], accounting for half of the reported *Lycoris* species. No tetranucleotide repetition was detected in all reported *Lycoris* species.

### 3.3. Statistics of Codon Usage

**Figure 3:**
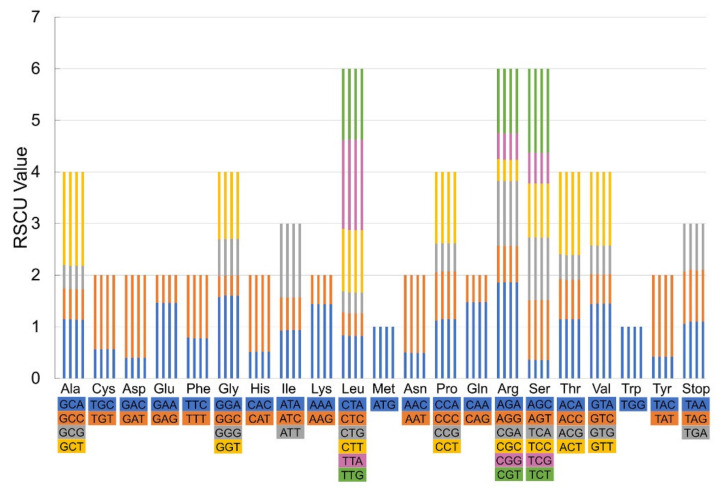
Codon content and codon usage of 20 amino acids and stop codons in protein-coding genes of the four *Lycoris* cp genomes. Each histogram from left to right was *L. incarnata*, *L. shaanxiensis*, *L. straminea*, and *L. houdyshelii*, respectively.

Codon usage analysis is beneficial for studies of evolution and new gene mining, which varies among different species [45]. Here, the complete cp genome sequences of four *Lycoris* were analyzed to investigate the amino acid frequency, the number of codon usage, the bias of codon usage, and relative synonymous codon usage (RSCU) (Table S3). Although the total number of codons was ranging from 48,207 to 49,641 in four species, showing a tiny change, the types of codons and amino acids were the same. A total of 64 codons were deduced, which were encoding 21 amino acids. Met and Trp were encoded by one codon usage, while others were encoding by multiple synonymous codons, ranging from two to six (Figure 3). The three highest frequency (AGA, GCT, and TTA) and four lowest frequency (AGC, GGC, GAC, and CTG) codons were observed in four species. It was defined as preferred codon usage when the RSCU value was >1.00 and vice versa. Except for methionine and tryptophan, there were 32 preferred and 30 non-preferred codon usages in *L. incarnata*, *L. shaanxiensis*, and *L. houdyshelii*, which was the same with the reported five *Lycoris* species, suggesting the main pattern of this codon usage in *Lycoris* [25]. In *L. straminea*, 31 preferred and 31 non-preferred codon usages were identified, which is different from any reported *Lycoris* species. The result will help us to understand the related patterns in *Lycoris* species and improve the research on codon usage in plant biology.

### 3.4. Inverted Repeats Contraction, Expansion, and Interspecific Comparison

**Figure 4:**
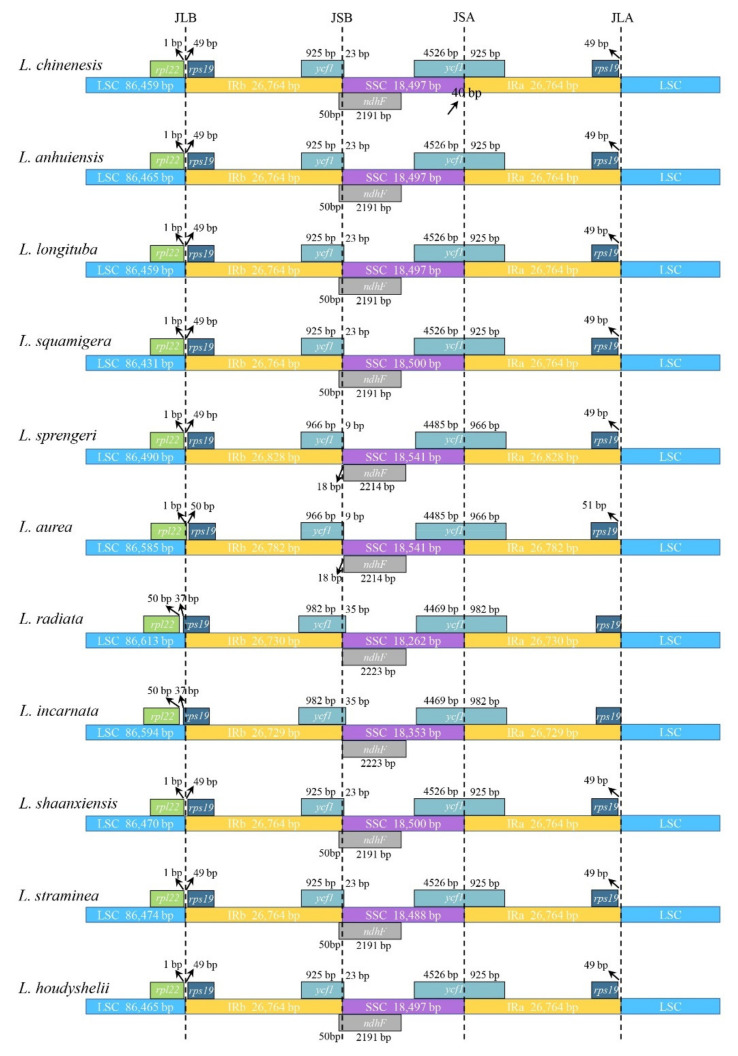
Comparison of border pattern of large single-copy regions (LSC), small single-copy regions (SSC), and an inverted repeat (IR) among 11 *Lycoris* chloroplast genomes.

The typical circular structure of the chloroplast genome consists of regions of IR, LSC, and SSC, which makes four boundaries (IRb/LSC, IRb/SSC, IRa/SSC, and IRa/LSC). The contraction or expansion of the IR regions commonly leads to the length variation of the chloroplast genomes among different plant species [20,46,47]. In the present study, we compared the IR/SC borders and the adjacent genes among the eleven *Lycoris* species, including the previously reported seven species [25,48,49] and four newly sequenced species. It showed the well-conserved genomic structure, but it also exhibited divergence at the IR/SC boundary regions among eleven *Lycoris* chloroplast genomes (Figure 4). In the most monocot plastid genome structure, IR regions expand into *rps19* [47]; there was no obvious expansion at the IRb/LSC boundary in *Lycoris*, except for the *L. radiata* and *L. incarnata*, their IRb regions expanded by 37 bp toward the *rps19* gene. In all *Lycoris* species, the IRa/SSC border extended into the *ycf1* genes with 925–982 bp. In addition, the *ndhF* gene overlapped with the IRa/SSC border by 50 bp in seven species, including *L. chinensis*, *L. anhuiensis*, *L. longituba*, *L. squamigera*, *L. shaanxiensis*, *L. straminea*, and *L. houdyshelii* (Figure 4).

**Figure 5:**
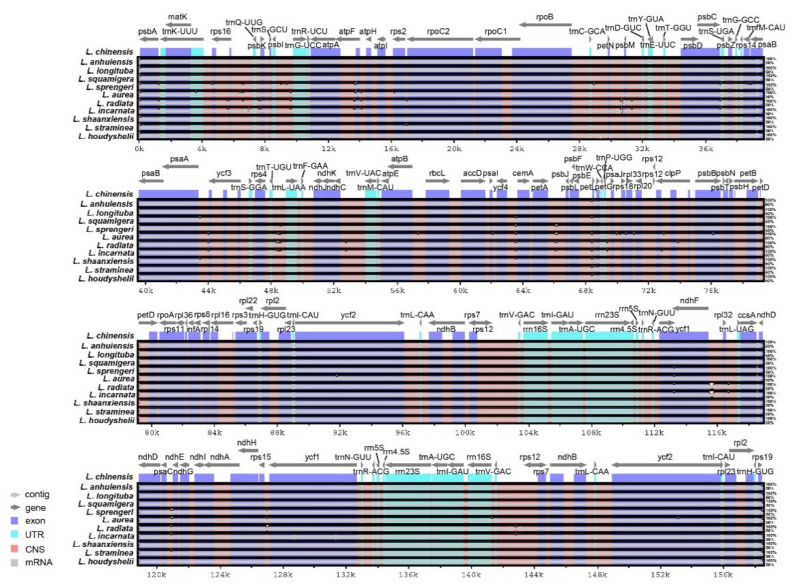
Sequence identity plot using mVISTA based on the complete cp sequences of 11 *Lycoris* species with *L. sprengeri* as a reference. A 70% cut-off identity was used for the plots, and the Y-scale represents the percent identity from 50% to 100%.

To rapidly identify the conserved sequences in long alignments, global interspecific comparisons were performed using software mVISTA [31,50]. A total of 11 *Lycoris* cp genome sequences (same as IR analysis) were selected for comparative analysis. Most of the sequence variations were found in the LSC and SSC regions, which being largely consistent with our previous studies [25], in which the IR regions showed the high sequence conservation of the 11 species (Figure 5). A lot of evidence indicated that *ndh*F has great power in discrimination at the low taxonomic level [51]. In *Lycoris*, *ycf1* and *ndhF* presented the most divergence in all species, suggesting the potential molecular markers for phylogenetic analysis and species identification. Although the length and boundary distribution characteristics of *ndh*F suggested that the *L. radiata* was a putative female parent of *L. incarnata*, the comparative analysis suggested the closer relationship of seven related species that have similar IR boundaries features (Figure 4 and Figure 5).

### 3.5. Phylogenetic Analysis

**Figure 6:**
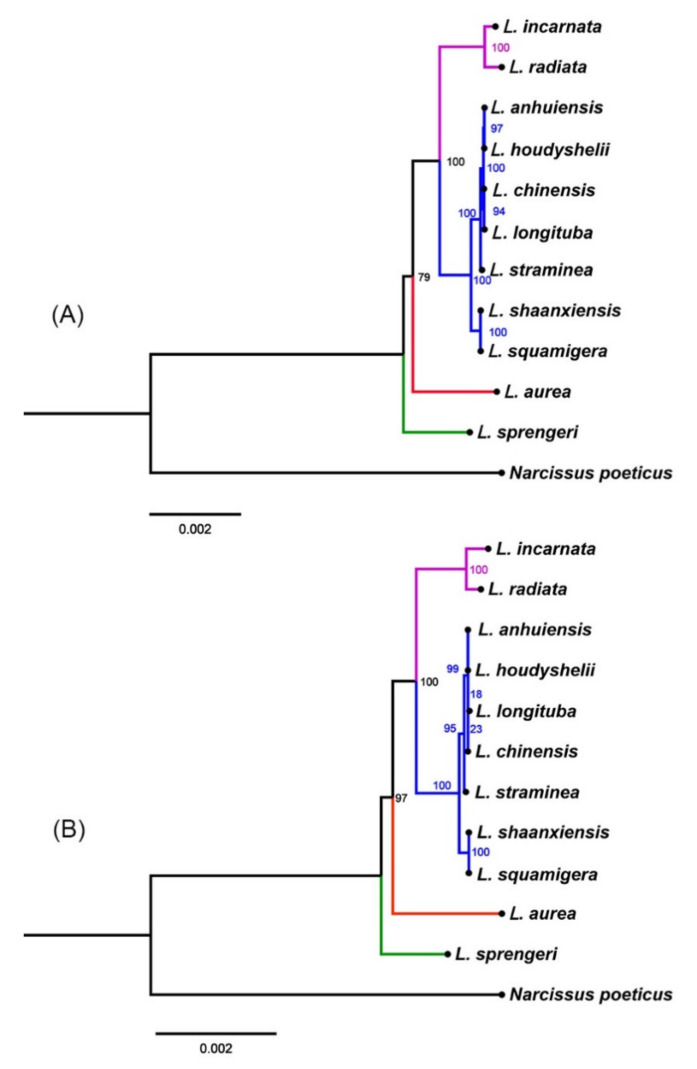
Phylogenetic analysis of the 11 *Lycoris* species by maximum likelihood (ML) analyses, *Narcissus poeticus* was the outgroup taxa. (**A**) The topology was constructed by the complete cp genome sequences. (**B**) The tree was constructed using 85 common protein-coding genes.

To explore the interspecific relationship and phylogeny reconstruction, a total of 11 *Lycoris* species with complete chloroplast genome sequences were selected for the construction of the maximum likelihood (ML) tree and *Narcissus poeticus* was chosen as the outgroup taxa. Both the complete plastid genome sequences and 79 common protein-coding genes were used for phylogenetic analysis, and the phylogenetic trees based on these two datasets showed the same topology (Figure 6).

Here, 11 *Lycoris* species were clustered into three main groups, and *L. sprengeri* is basal for the other species in *Lycoris*; however, previous cpDNA sequences analysis showed that the *L. radiata* had a basal position within *Lycoris* [10]. It involves the discussion of the origin of hybrid species of *Lycoris*. Some studies have suggested that the four species of *L. incarnata*, *L. shaanxiensis*, *L. straminea*, and *L. houdyshelii* were hybrid origin species [52], plastid DNA sequences and SCoT analysis showed that natural hybrids *L. incarnata* and *L. squamigera* were located in same clade [12,14]. Here, two species, *L. incarnata* and *L. shaanxiensis*, with similar morphological characteristics, are not clustered together. *L. incarnata* was clustered with *L. radiata*, suggesting that the *L. radiata* may be the female donor of the *L. incarnata. L. shaanxiensis* showed the closest relationship to *L. squamigera*, which was also considered as a hybrid origin species [14], suggesting the same ancestor of these two species, and *L. radiata* may be their donor according to the phylogenetic analysis by complete cp genome sequences. *L. straminea* and *L. houdyshelii* showed the most similar morphological and ecological characteristics except for the flower color. *L. straminea* was a species with multiple ecological properties, which always exhibits a color change from light yellow to medium yellow degrees, but the flower of *L. houdyshelii* is white. If only based on morphological features, *L. houdyshelii* could be considered as a variant; however, evidence has shown that they have a totally different chromosome number and karyotype [12,53]. RAPD analysis also indicated that they were clustered into two groups [9]. Evaluation of different methods, including morphology, karyotypes, plastid sequences, and molecular marker, produced both overlap and conflict on the interspecific relationship and phylogeny because of the variant resolutions and parameters.

Actually, the complete plastid sequence has been proved as an ideal method for phylogenetic relationship reconstruction. In the genus of *Lycoris*, some specific plastid gene sequences [14,54] and rDNA internal transcribed spacer (ITS) sequences [55,56] and have been developed before more complete cp genome sequences were available. More complete cp genome sequences provided adequate information and foundation for the clarification of inter-specific relationships and phylogenetic analysis. In the present study, we provided four cp genome sequences of *Lycoris*; they not only had similar morphological features but were also considered as the natural hybrid species. The phylogenic analysis supported the same group between *L. straminea* and *L. houdyshelii*, but *L. houdyshelii* showed a closer relationship with *L. anhuiensis, L. chinensis*, and *L. longituba* than *L. straminea*, the closest relationship of these five species also suggesting that *L. straminea* and *L. houdyshelii* may be derived from one of the three species.

## 4. Conclusions

In this study, we provided the complete cp genome sequences of *L. incarnata*, *L. shaanxiensis*, *L. straminea*, and *L. houdyshelii* and performed the interspecific comparison and phylogenetic analysis using whole cp genome sequences of 11 *Lycoris* species. The results not only showed the sequence conservation of genome size, gene number, and order but also distinguish the difference between IR-SC boundary regions. The interspecific comparison analysis supported the branch of phylogenetic analysis, where the species on the same sub-branch had the same border patterns, suggesting the high resolution and reliability of phylogenetic reconstruction by the complete cp genome sequences in *Lycoris*. Phylogeny analysis suggested that the *L. radiata* may be the female donor of the *L. incarnata*, *L. shaanxiensis*, and *L. squamigera*. *L. straminea* and *L. houdyshelii* may be derived from *L. anhuiensis*, *L. chinensis*, or *L. longituba*. The results will help to make the intraspecific relationship and evolution clear and benefit the identification, protection, and utilization of *Lycoris* germplasm resources.

## Figures and Tables

**Table 1:** Summary information of four chloroplast genomes of the *Lycoris* species.

Genome Features	*L. incarnata*	*L. shaanxiensis*	*L. straminea*	*L. houdyshelii*
Average organelle coverage	7291×	6761×	5531×	4581×
Genome size (bp)	158,405	158,498	158,490	158,490
LSC size (bp)	86,593	86,469	86,473	86,464
SSC size (bp)	18,352	18,499	18,487	18,496
IR size (bp)	26,730	26,765	26,765	26,765
GC content (%)	37.8	37.8	37.8	37.8
No. of genes	113	113	113	113
No. of PCGs	79	79	79	79
No. of tRNAs	30	30	30	30
No. of rRNAs	4	4	4	4
Duplicated genes	20	20	20	20

**Table 2:** Gene composition of four *Lycoris* chloroplast genomes.

Category of Genes	Group of Genes	Name of Genes
Genes for photosynthesis	Subunits of photosystem I	*psaB*, *psaA*, *psaI*, *psaJ, psaC*, *ycf4*
	Subunits of photosystem II	*psbA, psbK, psbI, psbM, psbD, psbC, psbZ, psbJ, psbL, psbF, psbE, psbB, psbT, psbN, psbH, ycf3*
	Subunits of NADH-dehydrogenase	*ndhJ, ndhK, ndhC, ndhB ^a^* (×2)*, ndhF, ndhD, ndhE,ndhG, ndhI, ndhA ^a^, ndhH*
	Subunits of cytochrome b/f complex	*petN, petA, petL, petG, petB ^a^, petD ^a^*
	Subunits of ATP synthase	*atpA, atpF ^a^, atpH, atpI, atpE, atpB*
	Subunit of rubisco	*rbcL*
Self-replication	Large subunit of ribosome	*rpl33, rpl20, rpl36, rpl14, rpl16 ^a^, rpl22, rpl2 ^a^ (×2), rpl23 (×2), rpl32*
	DNA dependent RNA polymerase	*rpoC2, rpoC1 ^a^, rpoB, rpoA*
	Small subunit of ribosome	*rps16 ^a^, rps2, rps14, rps4, rps18, rps12 ^b^ (×2), rps11, rps8, rps3, rps19* (×2)*, rps7* (×2)*, rps15*
	Ribosomal RNAs	*rrn16* (×2)*, rrn23* (×2)*, rrn4.5* (×2)*, rrn5* (×2)
	Transfer RNAs	*trnK-UUU ^a^, trnQ-UUG, trnS-GCU, trnG-GCC ^a^, trnR-UCU,trnC-GCA, trnD-GUC, trnY-GUA, trnE-UUC, trnT-GGU,trnS-UGA, trnG-GCC, trnfM-CAU, trnS-GGA, trnT-UGU,trnL-UAA ^a^, trnF-GAA, trnV-UAC ^a^, trnM-CAU, trnW-CCA,trnP-UGG, trnH-GUG* (×2)*, trnI-CAU* (×2)*, trnL-CAA* (×2)*,trnV-GAC* (×2), *trnI-GAU ^a^* (×2)*, trnA-UGC ^a^* (×2), *trnR-ACG* (×2)*trnN-GUU* (×2)*, trnL-UAG*
Other genes	Subunit of Acetyl-CoA-carboxylase	*accD*
	c-type cytochrome synthesis gene	*ccsA*
	Envelop membrane protein	*cemA*
	Protease	*clpP*
	Translational initiation factor	*infA*
	Maturase	*matK*
	Component of TIC complex	*ycf1* (x2)
Unknown	Conserved open reading frames	*ycf2* (x2)

^a^ means the genes containing a single intron; ^b^ indicates the genes containing two introns; (×2) indicates the genes duplicated in the IR regions.

## Data Availability

The data presented in this study are openly available at https://www.ncbi.nlm.nih.gov/ (accessed on 14 January 2021) with the access numbers of MW477439, MW477440, MW477441, and MW477442.

## Supplementary Materials

The following are available online at https://www.mdpi.com/article/10.3390/biology10080715/s1, Table S1: Splitting genes with introns and exons in the four *Lycoris* chloroplast genomes, Table S2: Chloroplast simple sequence repeats (cpSSRs) of four *Lycoris* species, Table S3: Relative synonymous codon usage (RSCU) in the four *Lycoris* chloroplast genomes.
