# Supplementary material for: Comparative Chloroplast Genomes of Four Lycoris Species (Amaryllidaceae) Provides New Insight into Interspecific Relationship and Phylogeny
Source: Biology (Basel). 2021 Jul 27;10(8):715. doi: 10.3390/biology10080715 (PMC8389210; doi:10.3390/biology10080715)
Supplement: Supplementary file 1 [file biology-10-00715-s001.zip › Table S1.pdf]

Table S1. Splitting genes with introns and exons in the four *Lycoris* chloroplast genomes.

**Species**	**Gene**	**Location**	**Start**	**End**	**ExonI (bp)**	**IntronI (bp)**	**ExonII (bp)**	**IntronII (bp)**	**ExonIII (bp)**
L. incarnata	*trnK-UUU*	LSC	1384	4045	46	2581	35		
	rps16	LSC	4640	5777	40	889	209		
	trnG-UCC	LSC	9803	10563	23	690	48		
	atpF	LSC	12496	13992	154	1086	257		
	rpoC1	LSC	21403	24210	432	750	1626		
	ycf3	LSC	44076	46065	126	725	231	755	153
	trnL-UAA	LSC	49044	49641	35	513	50		
	trnV-UAC	LSC	54027	54686	39	584	37		
	clpP	LSC	72574	74637	71	788	291	664	250
	petB	LSC	77527	78958	6	784	642		
	petD	LSC	79172	80430	8	737	514		
	rpl16	LSC	83880	85252	9	965	399		
	rpl2	IRA	87095	88581	391	665	428		
	ndhB	IRA	97745	99976	775	699	758		
	trnI-GAU	IRA	105524	106535	37	940	35		
	trnA-UGC	IRA	106600	107488	39	815	35		
	ndhF	IRA/SSC	113217	115546	2184	89	57		
	ndhA	SSC	123024	125246	559	1125	539		
	trnA-UGC	SSC	137512	138400	39	815	35		
	trnI-GAU	SSC	138465	139476	37	940	35		
	ndhB	SSC	145024	147255	775	699	758		
	rpl2	SSC	156422	157905	391	665	428		
L. shaanxiensis	*trnK-UUU*	LSC	1403	4060	46	2577	35		
	rps16	LSC	4656	5787	40	883	209		
	trnG-UCC	LSC	9809	10570	23	691	48		
	atpF	LSC	12495	13974	154	1069	257		
	rpoC1	LSC	21387	24194	432	750	1626		
	ycf3	LSC	44051	46040	126	725	231	755	153
	trnL-UAA	LSC	48982	49578	35	512	50		
	trnV-UAC	LSC	53977	54636	39	584	37		
	clpP	LSC	72541	74597	71	788	291	657	250
	petB	LSC	77486	78919	6	786	642		
	petD	LSC	79133	80391	8	737	514		
	rpl16	LSC	83841	85214	9	966	399		

	rpl2	IRA	87057	88541	391	666	428		
	ndhB	IRA	97708	99939	775	699	758		
	trnI-GAU	IRA	105492	106503	37	940	35		
	trnA-UGC	IRA	106568	107456	39	815	35		
	ndhA	SSC	123026	125248	559	1125	539		
	trnA-UGC	IRB	137513	138401	39	815	35		
	trnI-GAU	IRB	138466	139477	37	940	35		
	ndhB	IRB	145030	147261	775	699	758		
	rpl2	IRB	156428	157912	391	666	428		
L. straminea	*trnK-UUU*	LSC	1403	4060	46	2577	35		
	rps16	LSC	4657	5789	40	884	209		
	trnG-UCC	LSC	9809	10570	23	691	48		
	atpF	LSC	12495	13975	154	1070	257		
	rpoC1	LSC	21390	24198	432	751	1626		
	ycf3	LSC	44062	46051	126	725	231	755	153
	trnL-UAA	LSC	49009	49605	35	512	50		
	trnV-UAC	LSC	54000	54659	39	584	37		
	clpP	LSC	72545	74601	71	788	291	657	250
	petB	LSC	77491	78923	6	785	642		
	petD	LSC	79137	80395	8	737	514		
	rpl16	LSC	83845	85218	9	966	399		
	rpl2	IRA	87061	88548	391	666	428		
	ndhB	IRA	97712	99943	775	699	758		
	trnI-GAU	IRA	105496	106507	37	940	35		
	trnA-UGC	IRA	106572	107460	39	815	35		
	ndhA	SSC	123016	125238	559	1125	539		
	trnA-UGC	IRB	137505	138393	39	815	35		
	trnI-GAU	IRB	138458	139469	37	940	35		
	ndhB	IRB	145022	147253	775	699	758		
	rpl2	IRB	156420	157904	391	666	428		
L. houdyshelii	*trnK-UUU*	LSC	1403	4060	46	2577	35		
	rps16	LSC	4656	5789	40	885	209		
	trnG-UCC	LSC	9809	10570	23	691	48		
	atpF	LSC	12495	13974	154	1069	257		
	rpoC1	LSC	21384	24191	432	750	1626		
	ycf3	LSC	44053	46042	126	725	231	755	153

trnL-UAA	LSC	49000	49596	35	512	50		
trnV-UAC	LSC	53990	54649	39	584	37		
clpP	LSC	72535	74592	71	788	291	658	250
petB	LSC	77482	78914	6	785	642		
petD	LSC	79128	80386	8	737	514		
rpl16	LSC	83836	85209	9	966	399		
rpl2	IRA	87052	88536	391	666	428		
ndhB	IRA	97703	99934	775	699	758		
trnI-GAU	IRA	105487	106498	37	940	35		
trnA-UGC	IRA	106563	107451	39	815	35		
ndhA	SSC	123014	125236	559	1125	539		
trnA-UGC	IRB	137505	138393	39	815	35		
trnI-GAU	IRB	138458	139469	37	940	35		
ndhB	IRB	145022	147253	775	699	758		
rpl2	IRB	156420	157904	391	666	428		
