# Supplementary material for: Comparative Chloroplast Genomes of Four Lycoris Species (Amaryllidaceae) Provides New Insight into Interspecific Relationship and Phylogeny
Source: Biology (Basel). 2021 Jul 27;10(8):715. doi: 10.3390/biology10080715 (PMC8389210; doi:10.3390/biology10080715)
Supplement: Supplementary file 1 [file biology-10-00715-s001.zip › Table S2.pdf]

Table S2. Chloroplast simple sequence repeats (cpSSRs) of four *Lycoris* species

Species	SSR nr.	SSR type	SSR	Size (bp)	Start	End	Region
L. incarnata	1	p1	(A)13	13	3577	3589	LSC
	2	p1	(T)10	10	3700	3709	LSC
	3	p1	(A)10	10	7471	7480	LSC
	4	p1	(T)12	12	10609	10620	LSC
	5	c	(A)11...(T)10	27	14139	14165	LSC
	6	p1	(T)13	13	17009	17021	LSC
	7	c	(T)10...(T)10...(T)11	139	18965	19103	LSC
	8	p1	(T)13	13	23124	23136	LSC
	9	p1	(A)10	10	29978	29987	LSC
	10	p1	(A)10	10	30543	30552	LSC
	11	p1	(T)10	10	30976	30985	LSC
	12	p1	(T)10	10	31603	31612	LSC
	13	p1	(A)11	11	33173	33183	LSC
	14	p1	(A)12	12	33462	33473	LSC
	15	p1	(A)10	10	33770	33779	LSC
	16	p2	(TA)6	12	37237	37248	LSC
	17	p1	(A)10	10	37732	37741	LSC
	18	p1	(A)11	11	46906	46916	LSC
	19	c	(T)12...(T)12	54	48389	48442	LSC
	20	p1	(T)11	11	52900	52910	LSC
	21	p2	(TA)7	14	57415	57428	LSC
	22	p1	(T)10	10	59799	59808	LSC
	23	p1	(A)10	10	63788	63797	LSC
	24	c	(A)10...(T)10...(A)10	120	68328	68447	LSC
	25	p1	(T)11	11	69116	69126	LSC
	26	p1	(T)14	14	70217	70230	LSC
	27	p1	(T)11	11	71101	71111	LSC
	28	c	(A)11...(A)11	26	72895	72920	LSC
	29	p1	(T)13	13	73192	73204	LSC
	30	p1	(A)11	11	73978	73988	LSC
	31	p1	(T)10	10	76625	76634	LSC
	32	p1	(G)10	10	78283	78292	LSC
	33	p1	(A)11	11	80556	80566	LSC
	34	p1	(A)10	10	103198	103207	IRA
	35	p1	(T)15	15	115563	115577	SSC
	36	p1	(A)12	12	115844	115855	SSC
	37	p3	(ATT)6	18	116668	116685	SSC
	38	p1	(A)18	18	120864	120881	SSC
	39	c	(T)10...(T)11	98	126956	127053	SSC
	40	c	(T)11...(T)11	74	128106	128179	SSC
	41	p1	(A)10	10	130561	130570	SSC
	42	p1	(T)10	10	141793	141802	IRB

L. shaanxiensis	1	p1	(A)10	10	3596	3605	LSC
	2	p1	(T)10	10	4644	4653	LSC
	3	p1	(A)10	10	7488	7497	LSC
	4	p1	(T)11	11	10616	10626	LSC
	5	p1	(T)18	18	16988	17005	LSC
	6	c	(T)10...(T)10...(T)11	139	18949	19087	LSC
	7	p1	(T)13	13	23108	23120	LSC
	8	p1	(T)11	11	27878	27888	LSC
	9	p1	(A)10	10	28594	28603	LSC
	10	p1	(A)11	11	29338	29348	LSC
	11	p1	(A)10	10	30536	30545	LSC
	12	p1	(T)10	10	30969	30978	LSC
	13	p1	(A)11	11	33179	33189	LSC
	14	p1	(A)11	11	33468	33478	LSC
	15	p1	(T)10	10	36909	36918	LSC
	16	p1	(A)10	10	37729	37738	LSC
	17	p1	(A)10	10	46866	46875	LSC
	18	p1	(T)15	15	48347	48361	LSC
	19	p1	(A)12	12	50268	50279	LSC
	20	p1	(T)12	12	52849	52860	LSC
	21	p2	(TA)7	14	57365	57378	LSC
	22	p1	(A)10	10	61516	61525	LSC
	23	p1	(A)10	10	63755	63764	LSC
	24	p1	(T)10	10	68007	68016	LSC
	25	p1	(T)10	10	69076	69085	LSC
	26	p1	(T)15	15	70179	70193	LSC
	27	p1	(T)10	10	71068	71077	LSC
	28	p1	(T)10	10	73155	73164	LSC
	29	p1	(A)11	11	73938	73948	LSC
	30	p1	(A)10	10	83750	83759	LSC
	31	p1	(A)10	10	103166	103175	IRA
	32	p1	(T)10	10	115442	115451	SSC
	33	p1	(A)12	12	115852	115863	SSC
	34	p1	(T)10	10	116348	116357	SSC
	35	p3	(ATT)5	15	116677	116691	SSC
	36	p1	(A)13	13	120868	120880	SSC
	37	c	(T)10...(T)10	97	126958	127054	SSC
	38	c	(T)10...(T)11	74	128107	128180	SSC
	39	p1	(A)11	11	130562	130572	SSC
	40	p1	(T)10	10	141794	141803	IRB
L. straminea	1	p1	(A)10	10	3596	3605	LSC
	2	p1	(T)11	11	4644	4654	LSC
	3	p1	(T)10	10	5294	5303	LSC
	4	p1	(T)11	11	10616	10626	LSC

	5	p1	(T)20	20	16989	17008	LSC
	6	c	(T)10...(T)10...(T)11	139	18952	19090	LSC
	7	p1	(T)14	14	23111	23124	LSC
	8	p1	(T)12	12	27882	27893	LSC
	9	p1	(A)10	10	28599	28608	LSC
	10	p1	(A)10	10	29343	29352	LSC
	11	p1	(A)10	10	30540	30549	LSC
	12	p1	(T)11	11	30973	30983	LSC
	13	p1	(T)10	10	31615	31624	LSC
	14	p1	(A)11	11	33185	33195	LSC
	15	p1	(A)10	10	33474	33483	LSC
	16	p1	(T)10	10	36914	36923	LSC
	17	p1	(A)11	11	37734	37744	LSC
	18	p1	(A)10	10	46892	46901	LSC
	19	p1	(T)16	16	48373	48388	LSC
	20	p1	(A)11	11	50295	50305	LSC
	21	p2	(TA)7	14	57388	57401	LSC
	22	p1	(A)10	10	61540	61549	LSC
	23	p1	(A)10	10	63757	63766	LSC
	24	p1	(T)10	10	68009	68018	LSC
	25	p1	(A)10	10	68293	68302	LSC
	26	p1	(T)10	10	69079	69088	LSC
	27	p1	(T)15	15	70183	70197	LSC
	28	p1	(T)10	10	71072	71081	LSC
	29	p1	(A)10	10	72879	72888	LSC
	30	p1	(A)11	11	73942	73952	LSC
	31	p1	(T)10	10	76589	76598	LSC
	32	p1	(A)10	10	83754	83763	LSC
	33	p1	(A)10	10	103170	103179	IRA
	34	p1	(T)10	10	115446	115455	SSC
	35	p1	(A)12	12	115856	115867	SSC
	36	p1	(T)10	10	116352	116361	SSC
	37	p1	(A)12	12	120865	120876	SSC
	38	c	(T)11...(T)11	99	126948	127046	SSC
	39	c	(T)10...(T)11	74	128099	128172	SSC
	40	p1	(A)12	12	130553	130564	SSC
	41	p1	(T)10	10	141786	141795	IRB
L. houdyshelii	1	p1	(A)10	10	3596	3605	LSC
	2	p1	(T)10	10	4644	4653	LSC
	3	p1	(T)11	11	5293	5303	LSC
	4	p1	(T)11	11	10616	10626	LSC
	5	p1	(T)15	15	16988	17002	LSC
	6	c	(T)10...(T)10...(T)11	139	18946	19084	LSC
	7	p1	(T)13	13	23105	23117	LSC

8	p1	(T)12	12	27875	27886	LSC
9	p1	(A)10	10	28592	28601	LSC
10	p1	(A)10	10	30532	30541	LSC
11	p1	(T)10	10	30965	30974	LSC
12	p1	(T)10	10	31606	31615	LSC
13	p1	(A)11	11	33176	33186	LSC
14	p1	(A)10	10	33465	33474	LSC
15	p1	(T)10	10	36905	36914	LSC
16	p1	(A)11	11	37725	37735	LSC
17	p1	(A)10	10	46883	46892	LSC
18	p1	(T)16	16	48364	48379	LSC
19	p1	(A)10	10	50286	50295	LSC
20	p2	(TA)7	14	57378	57391	LSC
21	p1	(A)10	10	61529	61538	LSC
22	p1	(A)10	10	63746	63755	LSC
23	p1	(T)10	10	67998	68007	LSC
24	p1	(T)11	11	69067	69077	LSC
25	p1	(C)10	10	69829	69838	LSC
26	p1	(T)15	15	70173	70187	LSC
27	p1	(T)10	10	71062	71071	LSC
28	c	(A)10...(A)10	113	72869	72981	LSC
29	p1	(A)11	11	73933	73943	LSC
30	p1	(T)10	10	76580	76589	LSC
31	p1	(A)10	10	83745	83754	LSC
32	p1	(A)10	10	103161	103170	IRA
33	p1	(T)10	10	115437	115446	SSC
34	p1	(A)13	13	115847	115859	SSC
35	p1	(T)10	10	116344	116353	SSC
36	p1	(A)12	12	120857	120868	SSC
37	c	(T)11...(T)11	99	126948	127046	SSC
38	c	(T)10...(T)11	74	128099	128172	SSC
39	p1	(A)12	12	130553	130564	SSC
40	p1	(T)10	10	141786	141795	IRB
