# Supplementary material for: Comparative Chloroplast Genomes of Four Lycoris Species (Amaryllidaceae) Provides New Insight into Interspecific Relationship and Phylogeny
Source: Biology (Basel). 2021 Jul 27;10(8):715. doi: 10.3390/biology10080715 (PMC8389210; doi:10.3390/biology10080715)
Supplement: Supplementary file 1 [file biology-10-00715-s001.zip › Table S3.pdf]

Table S3. Relative synonymous codon usage (RSCU) in the four *Lycoris* chloroplast genomes.

Amino acid	Codon	Species							
		L. incarnata		*L. shaanxiensis*		*L. straminea*		*L. houdyshelii*	
		Number	RSCU	Number	RSCU	Number	RSCU	Number	RSCU
Ala	GCA	695	1.15	717	1.14	711	1.13	711	1.13
Ala	GCC	362	0.60	370	0.59	370	0.59	370	0.59
Ala	GCG	276	0.45	282	0.45	286	0.46	286	0.46
Ala	GCT	1094	1.80	1142	1.82	1142	1.82	1142	1.82
Cys	TGC	172	0.56	175	0.56	175	0.56	175	0.56
Cys	TGT	442	1.44	445	1.44	445	1.44	447	1.44
Asp	GAC	394	0.40	406	0.40	406	0.40	406	0.40
Asp	GAT	1584	1.60	1620	1.60	1620	1.60	1618	1.60
Glu	GAA	1906	1.46	1920	1.46	1922	1.46	1918	1.46
Glu	GAG	705	0.54	717	0.54	717	0.54	717	0.54
Phe	TTC	1104	0.79	1135	0.77	1133	0.77	1133	0.77
Phe	TTT	1703	1.21	1800	1.23	1799	1.23	1797	1.23
Gly	GGA	1217	1.58	1283	1.61	1281	1.61	1281	1.60
Gly	GGC	309	0.40	309	0.39	313	0.39	313	0.39
Gly	GGG	549	0.71	559	0.70	559	0.70	559	0.70
Gly	GGT	1005	1.31	1037	1.30	1035	1.30	1037	1.30
His	CAC	319	0.51	329	0.52	329	0.52	329	0.52
His	CAT	926	1.49	944	1.48	944	1.48	944	1.48
Ile	ATA	1239	0.93	1293	0.93	1295	0.93	1295	0.93
Ile	ATC	862	0.64	877	0.63	879	0.63	877	0.63
Ile	ATT	1916	1.43	1992	1.44	1992	1.43	1992	1.43
Lys	AAA	1912	1.44	1934	1.43	1928	1.43	1930	1.43
Lys	AAG	751	0.56	765	0.57	765	0.57	765	0.57
Leu	CTA	676	0.83	689	0.82	689	0.82	689	0.82
Leu	CTC	364	0.45	372	0.44	372	0.44	372	0.44
Leu	CTG	327	0.40	337	0.40	337	0.40	337	0.40
Leu	CTT	983	1.21	1017	1.21	1017	1.21	1017	1.21
Leu	TTA	1397	1.72	1475	1.76	1477	1.76	1477	1.76
Leu	TTG	1124	1.38	1153	1.37	1155	1.37	1157	1.37
Met	ATG	1103	1.00	1151	1.00	1149	1.00	1151	1.00
Asn	AAC	571	0.50	577	0.49	577	0.49	577	0.49
Asn	AAT	1714	1.50	1790	1.51	1790	1.51	1790	1.51
Pro	CCA	549	1.12	575	1.15	575	1.15	575	1.15
Pro	CCC	458	0.93	464	0.93	464	0.93	464	0.93
Pro	CCG	272	0.56	270	0.54	270	0.54	270	0.54
Pro	CCT	682	1.39	694	1.38	694	1.38	694	1.38
Gln	CAA	1274	1.47	1296	1.47	1302	1.48	1304	1.48
Gln	CAG	456	0.53	464	0.53	464	0.52	462	0.52

Arg	AGA	941	1.86	961	1.87	961	1.86	961	1.87
Arg	AGG	359	0.71	361	0.70	361	0.71	361	0.70
Arg	CGA	635	1.26	647	1.26	645	1.25	647	1.26
Arg	CGC	212	0.42	210	0.41	210	0.41	210	0.41
Arg	CGG	257	0.51	265	0.51	265	0.51	267	0.52
Arg	CGT	629	1.24	645	1.25	645	1.25	643	1.25
Ser	AGC	232	0.36	239	0.36	237	0.36	237	0.36
Ser	AGT	739	1.15	771	1.16	771	1.16	771	1.16
Ser	TCA	778	1.21	802	1.21	800	1.21	800	1.21
Ser	TCC	676	1.05	697	1.05	699	1.05	699	1.05
Ser	TCG	385	0.60	395	0.59	395	0.60	395	0.60
Ser	TCT	1046	1.63	1087	1.63	1089	1.63	1087	1.63
Thr	ACA	694	1.15	714	1.15	714	1.14	714	1.14
Thr	ACC	471	0.78	474	0.76	476	0.77	476	0.76
Thr	ACG	294	0.49	298	0.48	298	0.48	298	0.48
Thr	ACT	965	1.59	1007	1.62	1007	1.62	1007	1.61
Val	GTA	908	1.44	943	1.45	941	1.44	943	1.45
Val	GTC	360	0.58	366	0.56	366	0.57	366	0.57
Val	GTG	351	0.56	363	0.56	363	0.56	363	0.56
Val	GTT	900	1.43	932	1.43	932	1.43	932	1.43
Trp	TGG	878	1.00	901	1.00	898	1.00	898	1.00
Tyr	TAC	372	0.42	388	0.42	388	0.42	388	0.42
Tyr	TAT	1389	1.58	1453	1.58	1449	1.58	1453	1.58
Stop	TAA	122	1.06	128	1.10	128	1.10	128	1.10
Stop	TAG	114	1.01	115	1.01	113	0.99	115	1.01
Stop	TGA	108	0.94	104	0.89	106	0.91	104	0.89
